# An improved whole‐cell biotransformation system for (*S*)‐equol production

**DOI:** 10.1002/fsn3.2840

**Published:** 2022-03-21

**Authors:** Bing‐Juan Li, Meng‐Ying Xiao, Xin‐Yu Dong, Zhao‐Xiang Huang

**Affiliations:** ^1^ 12607 Tianjin Key Laboratory of Food and Biotechnology Department of Biotechnology and Food Science Tianjin University of Commerce Tianjin China

**Keywords:** (*S*)‐equol, daidzein, recombinant *Escherichia coli*, whole‐cell biotransformation

## Abstract

(*S*)‐equol, the most active metabolite of the soybean isoflavones in vivo, has exhibited various biological activities and clinical benefits. Existing studies on the heterologous biosynthesis of (*S*)‐equol via the engineered *E. coli* constructed have been significantly progressed. In the present study, the engineered *E. coli* was further improved to be more suitable for (*S*)‐equol production. The four enzymes involved in the biosynthesis of (*S*)‐equol and another GDH for NADPH regeneration were combined to construct the recombinant *E. coli* BL21(DE3). The optimal conditions for (*S*)‐equol production were explored, respectively. The yield of equol reached 98.05% with 1 mM substrate daidzein and 4% (wt/vol) glucose. Even when the substrate concentration increased to 1.5 mM, (*S*)‐equol could maintain a high yield of 90.25%. Based on the 100 ml one‐pot reaction system, (*S*)‐equol was produced with 223.6 mg/L in 1.5 h. The study presented a more suitable engineered *E. coli* for the production of (*S*)‐equol.

## Graphical abstract

The four enzymes involved in the biosynthesis of (*S*)‐equol and another GDH for NADPH regeneration were combined to construct the recombinant *E. coli* BL21(DE3).The engineered *E. coli* was improved to be more suitable for (*S*)‐equol production.

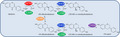

## 1 INTRODUCTION

Soybean and a wide range of soy products are recognized as the popular favorite food for their universality and nutritional value. (*S*)‐equol, one of the metabolites of the soybean isoflavones in vivo, has aroused huge attention. It can exert an excellent estrogenic activity by binding effectively to the type β‐estrogen receptor (Muthyala et al., 2004). It also shows the antioxidant 100 times higher than its precursor daidzein (Gou et al., 2015; Hwang et al., 2003). Besides, (*S*)‐equol has been proven to have antitumor and anti‐inflammatory activities (Bandara et al., 2010; Kim et al., 2014). Given its superior biological activities, (*S*)‐equol is capable of preventing menopausal symptoms without increasing the incidence of breast cancer, as well as preventing hormone‐dependent diseases (e.g., osteoporosis and prostate cancer) (Ozasa et al., 2004). As indicated from existing researches, (*S*)‐equol has broad clinical applications (Fatima et al., 2020; Lu et al., 2021; Mayo et al., 2019). However, only 25%–30% of young people can excrete (*S*)‐equol in vivo when supplied with soy foods (Rowland et al., 2018). Thus, an efficient method should be urgently developed for (*S*)‐equol production.

Since (*S*)‐equol was first detected from pregnant mare urine, (*S*)‐equol was gradually reported to be converted by intestinal microbes. Since most intestinal microbes pertain to anaerobic microorganisms, it was not until 2005 that the pure intestinal microbes were reported for the fermentation of (*S*)‐equol in vitro (Decroos et al., 2005). Otsuka Pharmaceutical generated the first nutraceutical containing natural (*S*)‐equol from *Lactococcus garvieae* (Ishiwata et al., 2009). Since then, more equol‐producing bacteria have been progressively identified from *Bacteroides*, *Streptococcus*, *Lactococcus*, *Slackia*, *Bifidobacterium*, *Enterococcus*, *Lactobacillus*, and *Eggerthella* (Guo et al., ; Kim et al., 2009; Matthies et al., 2009; Shimada et al., 2012; Tsuji et al., 2010).

The heterologous biosynthesis of (*S*)‐equol has been studied by several groups. The biosynthetic pathway of (*S*)‐equol from daidzein consists of a four‐step enzyme catalysis as previously elaborated, which comprises daidzein reductase (DZNR), dihydrodaidzein reductase (DHDR), tetrahydrodaidzein reductase (THDR), as well as dihydrodaidzein racemase (DDRC) (Schroder et al., 2013). Gao et al. (2016) initially constructed a recombinant *E. coli* that harbored the daidzein and genistein reductase gene (*dgr*) from *Slackia* sp. AUH‐JLC159. Besides, they successfully performed the aerobic biosynthesis of dihydrodaidzein (DHD) and dihydrogenistein (DHG). Lee et al. (2016) built the heterologous co‐expression of the four‐step enzyme process from *Slackia isoflavoniconvertens* in a recombinant *E.coli*. On that basis, they increased the (*S*)‐equol yield to 69.8 mg L^‐1^ h^‐1^ by exploiting the site directed mutation of DHDR with P212A in the recombinant *E.coli*. Furthermore, some efforts have been made to improve the stability of engineered strain and the production of equol.

**FIGURE 1:**
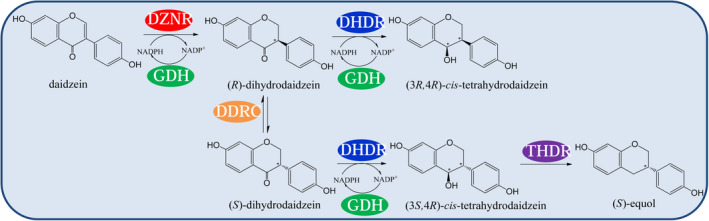
The constructed recombinant cell for the bioconversion of daidzein to (*S*)‐equol

It is noteworthy that two of the four‐step enzymes in the (*S*)‐equol biosynthetic pathway pertain to NADPH‐dependent oxidoreductase. In the recombinant cells, NADPH is continuously consumed as daidzein is transformed to (*S*)‐equol. In this study, a stable recombinant cell was constructed, which was capable of producing (*S*)‐equol and regenerating the consumed NADPH simultaneously. Moreover, multi‐enzyme co‐expression system was constructed by co‐expressing the (*S*)‐equol‐producing genes (i.e., *L‐ddrc*, *L‐dznr*, *L‐dhdr*, and *L‐thdr*) and the NADPH regenerating gene (*gdh*). It was stressed that the mentioned five enzymes were co‐expressed on one recombinant vector, so the recombinant cell turned out to be more stable and efficient. The catalytic mode of the whole cell is summarized in a schematic diagram (Figure 1).

## 2 MATERIAL AND METHODS

### 2.1 Materials and strains

The gene information is presented below: the four genes for (*S*)‐equol biotransformation from *Slackia isoflavoniconvertens*, including daidzein reductase (GenBank accession: AFV15453.1), dihydrodaidzein reductase (P212A mutant, proved to be more active (Lee et al., 2016)) (GenBank accession: AFV15451.1), tetrahydrodaidzein reductase (GenBank accession: AFV15450.1), hypothetical protein (proved to be dihydrodaidzein racemase, DDRC) (GenBank accession: AFV15447.1), and glucose dehydrogenase from *Bacillus megaterium* for NADPH regeneration (GenBank accession: AAA22475.1 (Hu et al., 2017)). All the genes were synthesized by complying with *E. coli* codon preference by RuiBiotech Crop (Beijing, China). Moreover, the restriction enzymes and the ligation kit were purchased from New England Biolabs (Ipswich, MA). *E. coli* JM109 and *E. coli* BL21(DE3) competent cells and plasmids originated from TransGen Biotech (Beijing, China). Daidzein was purchased from J&K Scientific Ltd (Beijing, China). (*S*)‐equol and (*R*)‐equol were provided by Daicel Chiral Technologies Co., Ltd. (Shanghai, China). Furthermore, Streptomycin and isopropyl‐β‐D‐thiogalactopyranoside (IPTG) were ordered from Sangon Biotech Bio (Shanghai, China). All other chemicals were of analytical grade unless otherwise indicated.

### 2.2 Recombinant strains construction and growth

To construct the five genes fusion, *dznr* and *gdh* were fused by the overlap PCR via a promoter sequence, and the fusion length was 2870 bp. The gene (i.e., *dhdr*, *thdr*, and *ddrc*) was fused by three rounds of the overlap PCR, the three genes were separated by using their respective T7 promoters (TAATACGACTCACTATA); *lac* operator (AATTGTGAGCGGATAACAATT) and *SD* sequences (AAGGAG). The fusion length was 3075 bp. Subsequently, the fragment *dznr*‐*gdh* was digested by *Bam*HI and *Not*I and then linked into multiple cloning site 1 (MCS1) of pCDFDuet‐1. The fragment *dhdr*+thdr+*ddrc* was digested with *Nde*I and *Xho*l and then linked into the multiple cloning site 2 (MCS2) of pCDFDuet‐1. The recombinant plasmid was transformed into *E. coli* JM109 for the sequence verification. The transformed colonies were selected on a Luria–Bertani (LB) agar (10.0 g/L tryptone, 5.0 g/L yeast extract, 10.0 g/L sodium chloride, and 15.0 g/L agar) plate containing streptomycin (50 μg/mL). The recombinant plasmid was transformed into *E. coli* BL21(DE3) after being sequenced.

*E. coli* BL21(DE3) cells harboring the recombinant plasmids were grown overnight at 37°C and 200 r.p.m. in 10‐ml LB medium containing streptomycin (50 μg/ml) within a shake flask. The overnight cultures were suspended in 100‐ml fresh LB medium at a final concentration of 1% (v/v), and then grown at 37°C and 200 r.p.m. for 2–3 h until OD600 reached 0.8. Next, 0.1 mM FeSO_4_ and different concentrations of isopropyl‐b‐D‐thiogalactopyranoside (IPTG) (i.e., 0.1, 0.5, and 1 mM) were added for the expressions of the recombinant enzymes. The cell cultures were incubated for an additional 12 h at 18°C and 150 r.p.m. The soluble expressions of the recombinant enzymes were detected with the SDS‐PAGE gels (12%) after the cells were disrupted by ultrasound, and followed by Coomassie Blue staining.

### 2.3 Whole‐cell biotransformation

The recombinant bacteria cells were harvested through the centrifugation at 5000 *g* for 5 min after the induction culture. Subsequently, the cells were washed with 200 mM potassium phosphate buffer (pH: 8.0) and then resuspended in the above buffer.

For test of the optimal temperature for the whole‐cell biotransformation, cell pellets were resuspended in 0.5 ml of reaction solution (OD600 = 20) containing 200 mM potassium phosphate buffer (pH: 8.0), 0.5 mM daidzein, and 2% glucose. The biotransformation was conducted at the temperatures (i.e., 28, 30, 32, 34, 36, and 38°C) for 3 h.

For test of the optimal pH for the whole‐cell biotransformation, cell pellets were resuspended in 0.5 ml of reaction solution (OD600 = 20) containing 0.5 mM daidzein and 2% glucose in 200 mM potassium phosphate buffer with pH 6.0, 6.5, 7.0, 7.5, and 8.0, as well as in 200 mM Tris‐HCl buffer with pH 8.5 and 9.0. The biotransformation was conducted at 30°C for 3 h.

The effects of the concentration of daidzein and glucose were explored in the following reaction system: cell pellets (OD600=20) were resuspended in 0.5 ml of 200 mM potassium phosphate buffer (pH 8.0), the concentration of daidzein was set to 0.5, 1, 1.5, and 2 mM, and the corresponding glucose concentration was set to 0, 2, 4, 6, 8 (wt/vol). The biotransformation was conducted at 32°C for 3 h.

The scale‐up reaction system was conducted under the optimal conditions with 1 mM daidzein, 4% (wt/vol) glucose, cell pellets (OD600 = 20) in 200 mM potassium phosphate buffer (pH: 8.0). Furthermore, the reaction volume was scaled up from 1 to 100 ml. An aliquot of 50‐μL sample solution was generally taken at the indicated times. The reactions were terminated by adding 50‐μL acetonitrile through the taking out of 50 μL for the further HPLC analysis.

### 2.4 High‐performance liquid chromatography (HPLC)

The reactions were terminated by adding acetonitrile. Subsequently, the supernatant was filtered via 0.22 μm filtration after the high‐speed centrifugation (13,000 rpm, 10 min). The samples were analyzed by using the HPLC [Cosmosil‐C18, flow rate: 1ml/min, column temperature: 28°C, UV detection wavelength: 280 nm, mobile phase: isocratic elution with acetonitrile (A): water (B)‐60:40(v/v)]. The conversion rate was determined as the product concentration /(substrate concentration)/100%. The product concentration or substrate concentration was calculated from the standard curve. Values were expressed as the means ± *SD* (*n* = 3).

The *e.e* values were analyzed by using the chiral HPLC that was connected to a CHIRALCEL OJ‐H column (Daicel Chemical Industries, Ltd., Tokyo, Japan). The mobile phase was n‐hexane: ethanol‐60:40 (v/v). The *e.e* values were determined with the formula 100%*(S‐R)/(S + R).

## 3 RESULTS

### 3.1 Constructed five enzymes in one plasmid for the individual expression

**FIGURE 2:**
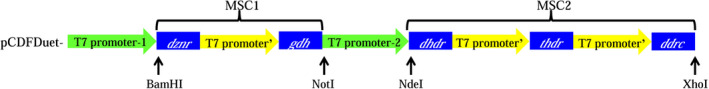
The gene expression pattern in the recombinant plasmid. (T7 promoter‐1, T7 promoter‐2, were the original fixed promoters on the plasmid pCDFDuet‐1. T7 promoter was added by fusion PCR)

First, the codon optimization of the five enzyme genes was conducted for their efficient expression in *E. coli*. To express five enzymes individually, the respective gene exhibited its own promoter and terminator. Besides, four rounds of overlap PCR were performed to achieve two fusion genes. The length of the fusion gene reached 2870 and 3075 bp. Lastly, the fusion gene was connected to the expression vector to generate a recombinant plasmid pCDFDuet‐*dznr‐gdh ‐dhdr‐thdr‐ddrc*. Figure 2 presents the gene expression pattern in the recombinant plasmid. For the protein expression, the verified recombinant plasmid was transformed into *E.coli* BL21(DE3). It was demonstrated that by regulating the concentration of streptomycin in the culture system, the recombinant plasmid was continuously stable in the cell.

**FIGURE 3:**
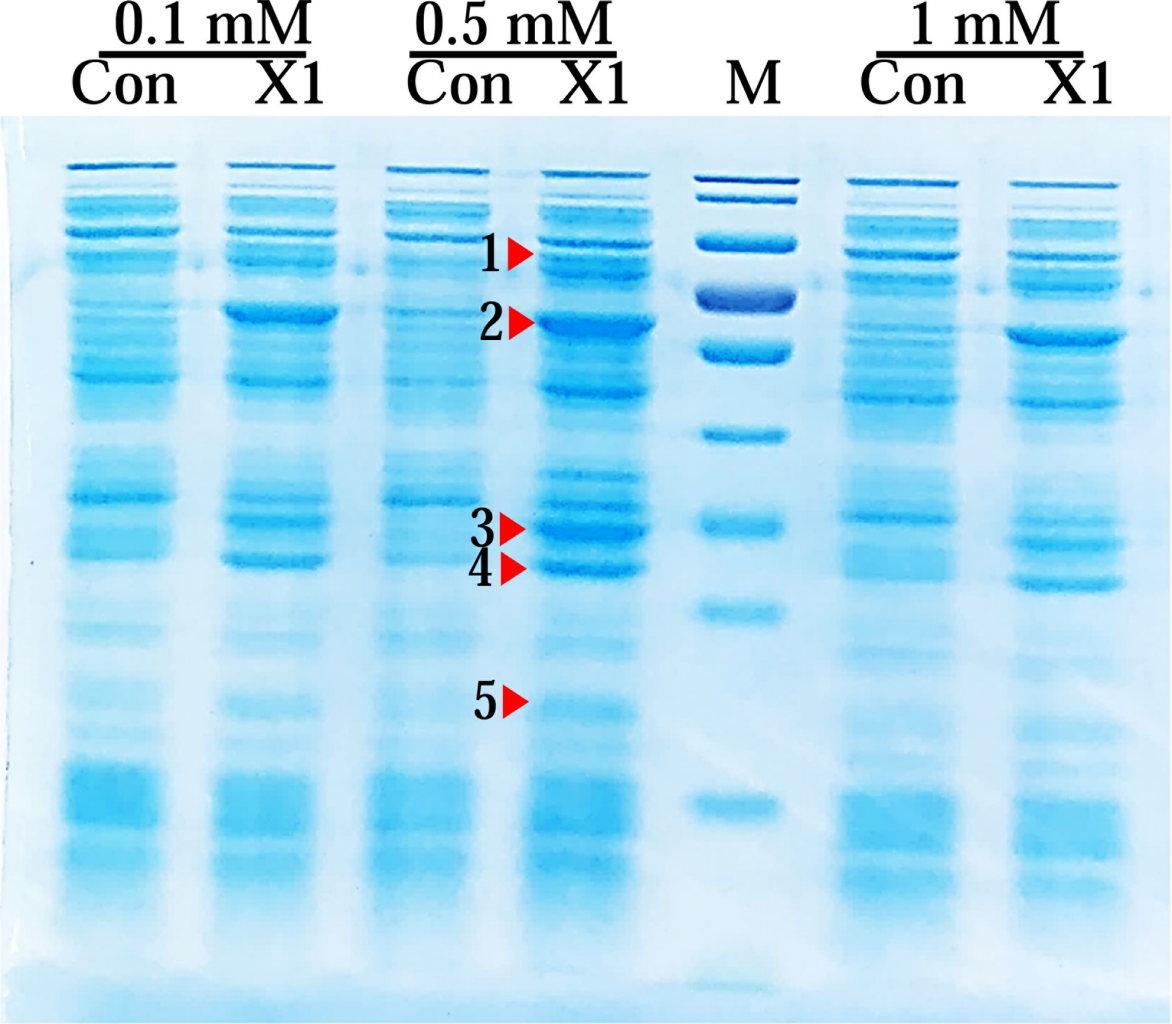
SDS‐PAGE analysis of the soluble expressions of recombinant proteins under different concentrations of IPTG. Con: Ultrasound supernatant of the recombinant strain with empty plasmid pCDFDuet after induced. X1: Ultrasound supernatant of the recombinant strain with recombinant plasmid pCDFDuet‐*dznr‐gdh‐dhdr‐thdr‐ddrc* after induced. 1. DZNR:76 kDa; 2. THDR:56 kDa; 3. DHDR:30.5k Da; 4. GDH:27 kDa; 5. DDRC: 18 kDa. M: Molecular weight standard (Molecular weight from top to bottom:170;130;100;70;55;40;35;25;15;10 (kDa))

After being induced with the isopropyl‐β‐D‐thiogalactopyranoside (IPTG), the expressions of recombinant proteins were detected by using the SDS‐PAGE (Figure 3). Compared with the control, the soluble expressions of all five enzyme proteins (i.e., DZNR, DDRC, DHDR, THDR, and GDH) were confirmed by using the SDS‐PAGE, and the optimal concentration of the IPTG was 0.5 mM.

### 3.2 The optimal whole cell activities for (*S*)‐equol production

**FIGURE 4:**
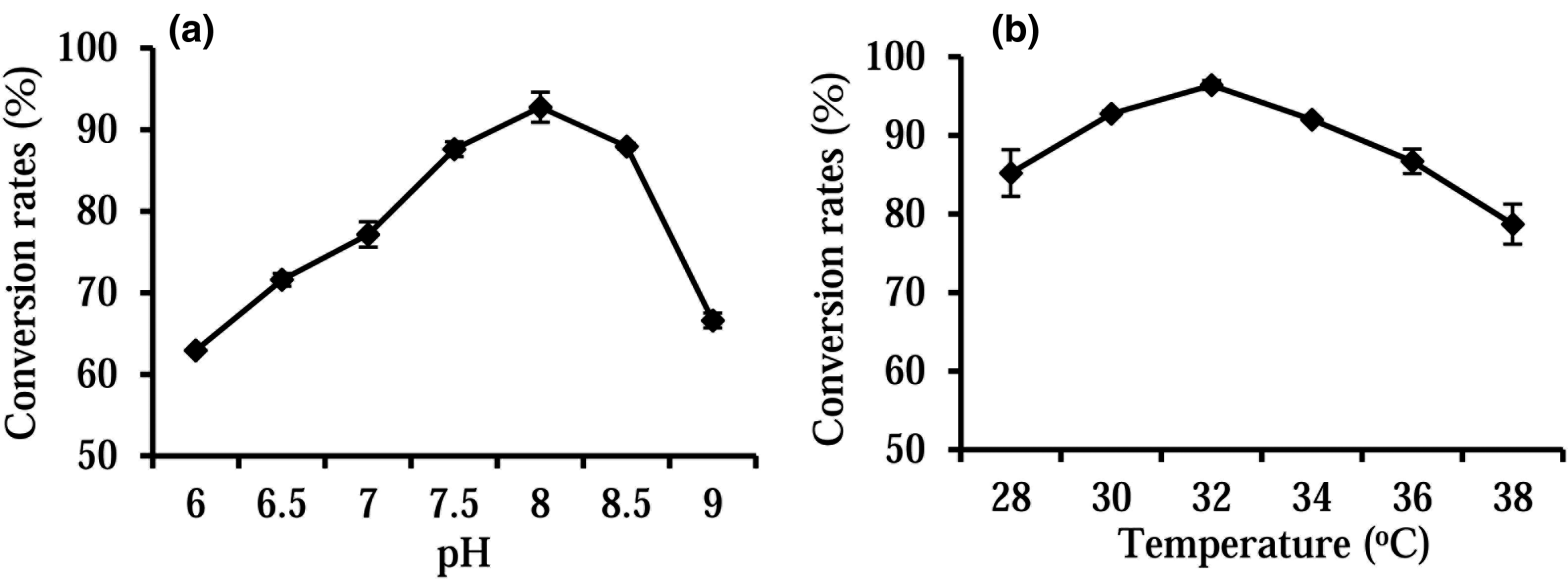
Effect of pH (a) or temperature (b) on whole cell activity. The data represent the mean ± *SD*, *n* = 3

Temperatures and pH values are vital factors of enzyme activities. Specific to the optimal catalytic conditions of the recombinant whole cell system, the balance of the activities of five enzymes (i.e., DZNR, DDRC, DHDR, THDR, and GDH) should be considered. Among the mentioned five enzymes, DZNR catalytic activity was reported to be vulnerable to oxygen or to high temperatures (Lee et al., 2016). Thus, a wide range of temperatures and pH values were examined, the results indicated that the optimal temperature for the whole cell transformation was 32°C (Figure 4a). The optimal pH values for the whole cell transformation reached 8.0 (Figure 4b), complying with the result in an earlier report.

**FIGURE 5:**
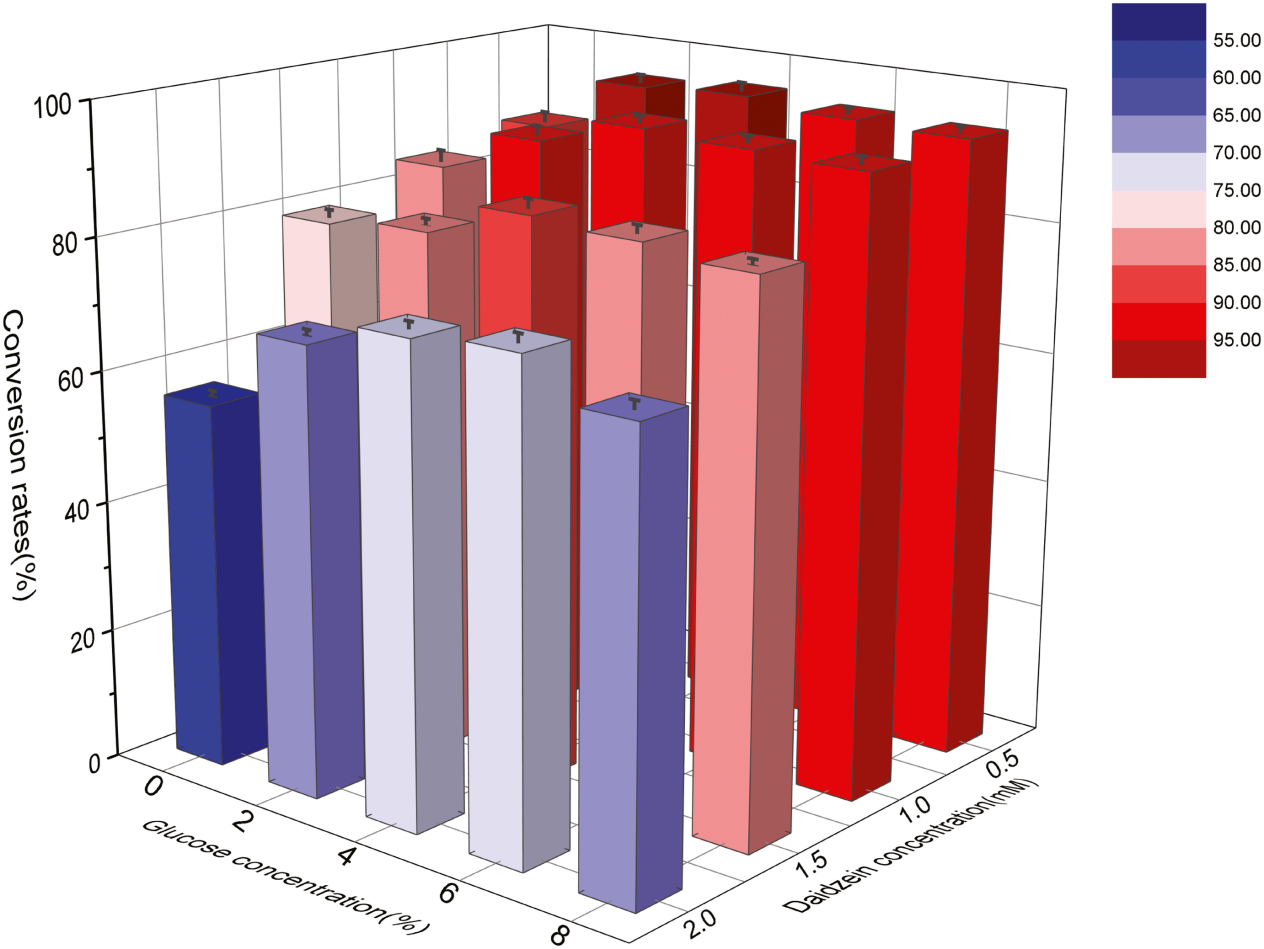
Effect of the concentration of substrate daidzein and glucose on the whole cell activities. The data represent the mean ± *SD*, *n* = 3

To further increase the production of (*S*)‐equol in the whole‐cell catalytic system, the effects of the concentration of substrate daidzein, glucose concentration were investigated. As indicated from the results (Figure 5), under the substrate concentration of 0.5 mM, the glucose concentration was 4%, and the yield of (*S*)‐equol in the transformation system reached the highest to 98.4%. Moreover, under the glucose concentration of 4%, the substrate concentration increased to 1mM, the yield of (*S*)‐equol still maintained a high conversion reaching 98.05%. It was reported that the (*S*)‐equol production would decrease with the increase in the substrate concentration over 1mM. This result was probably explained as the excessive production of (*S*)‐equol inhibited the reaction. This study confirmed that under the substrate concentration of 1.5 mM, the yield of (*S*)‐equol could achieve 90.25%. However, under the substrate concentration of 2.0 mM, the conversion dropped significantly.

### 3.3 Evaluated the catalytic efficiency of the recombinant *E.coli*

**FIGURE 6:**
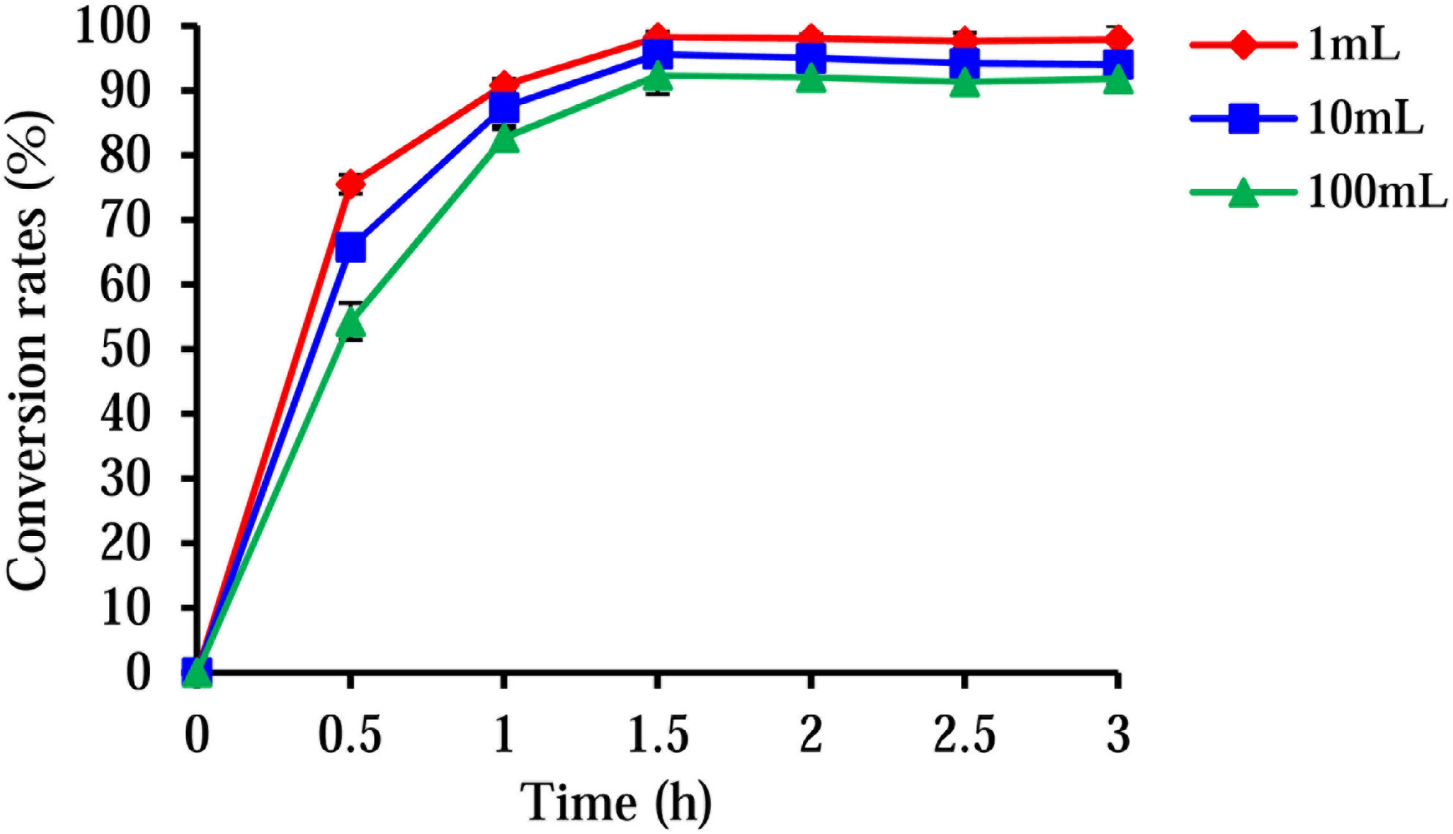
Time‐concentration curves of the conversion rates in the reactions. The data represent the mean ± *SD*, *n* = 3

The final concentration of the substrate concentration assigned was 1 mM, the glucose concentration was 4%, and the cell concentration was OD600=20, and the yield of (*S*)‐equol was detected in the 1 ml whole‐cell biotransformation system. The reaction reached the equilibrium after 1.5 h. The yield of (*S*)‐equol reached 182.9, 212.0, 237.9, 237.5, 236.5, and 237.1 mg/L at 0.5, 1, 1.5, 2, 2.5, and 3 h, respectively (Figure 6). All the *e.e* (enantiomeric excess) values reached over 99.0% (*S*).

The one‐pot reaction volume was amplified from 1 to 10 and 100 ml, and a similar yield of (*S*)‐equol was obtained, in which the (*S*)‐equol yield reached 231.6 and 223.6 mg/ml in the 10 and 100 ml reaction system, respectively, at 1.5 h. As revealed from the mentioned results, the constructed recombinant strain could maintain the catalytic activity in a certain transformation system.

## 4 DISCUSSION

A growing number of studies revealed that (*S*)‐equol exhibits various pharmacological activities. Given the excellent biological activity of (*S*)‐equol, an efficient synthesis method of (*S*)‐equol should be developed. The existing approaches for the production of (*S*)‐equol include total chemical synthesis of (*S*)‐equol, the natural bacteria fermentation, and the synthetic biology for (*S*)‐equol production. A great progress has been achieved in the chemical synthesis of equol. (*S*)‐equol was synthesized in >98% *e.e* with a good overall yield starting from soy isoflavones (Yang et al., 2012). However, there were some defects that cannot be ignored (e.g., application of organic solvents and tedious reaction conditions). With the number and scope of natural equol‐producing strains discovered, natural bacteria fermentation has been suggested as a promising approach for the future commercial and economical equol production (Kim et al., 2009; Matthies et al., 2009; Shimada et al., 2012; Tsuji et al., 2010). Nevertheless, most of the natural equol‐producing strains pertain to the strict anaerobes and grow at quite low rate, thereby limiting the large‐scale industrial fermentation for equol production.

By developing the synthetic biology, new opportunities can be created for the efficiency production of biologically active products. In existing researches, the four enzymes for equol transformation were clarified and verified, and the four enzyme genes for equol transformation were linked into two plasmids (Lee et al., 2016, 2017). There might be a risk of unbalanced plasmid copy number, so the stability of the strain was likely to be affected. In the present study, all of the enzyme genes were constructed in one plasmid, thereby causing the recombinant strain constructed in this study to be more stable than the previous ones. NADPH is an essential component for the biosynthesis of cellular components inside the cell, and it is also an important cofactor for producing a number of nutraceuticals and fine chemicals. Oxidation of glucose catalyzed by glucose dehydrogenase (GDH) is often used for NADPH regeneration (Lee et al., 2013). In the enzymatic conversion system, DZNR and DHDR require NADPH as the coenzyme to participate in the enzyme activity. So, the consumed NADPH could be restored by GDH, the balance of the redox state in the cell could be maintained. For this reason, the conversion of equol in this study could quickly reach equilibrium compared with the previous cells. The strain constructed here exhibited a higher transformation rate (13.5 mg L^‐1^ h^‐1^) and reached the equilibrium more quickly (1.5 hr with 1 mM daidzein in 100 ml) than the previous. In addition, some efforts have been tried in achieving high‐level expression of enzymes including optimizing the gene sequences of the five enzymes by codon optimization; each of these five genes contains their own upstream sequence and downstream sequence for controlling gene transcription and translation (Liu et al., 2013).

Besides, some efforts were proposed for improving (*S*)‐equol production. The yield of (*S*)‐equol could be further improved through the solvent engineering (increased to 1.22 g/L) or the pathway engineering (the recombinant strain increased the (*S*)‐equol resistance) (Lee et al., 2018; Li et al., 2018). Furthermore, the protein engineering and the immobilized cell technology will be adopted to further increase equol production. Moreover, the recombinant strain containing the four enzymes for equol production has been proven to be used for producing 5‐hydroxyequol. On the whole, this study laid a solid basis for the subsequent studies on the large‐scale preparation of (*S*)‐equol and derivatives.

## CONFLICT OF INTEREST

The authors declare that they have no competing interests.

## Data Availability

The data that support the findings of this study are available from the corresponding author upon reasonable request.

## References

[fsn32840-bib-0001] Bandara, M. , Arun, S. J. , Allanson, M. , Widyarini, S. , Chai, Z. , & Reeve, V. E. (2010). Topical isoflavonoids reduce experimental cutaneous inflammation in mice. Immunology and Cell Biology, 88(7), 727–733. 10.1038/icb.2010.26 20212509

[fsn32840-bib-0002] Bandara, M. , Arun, S. J. , Allanson, M. , Widyarini, S. , Chai, Z. , & Reeve, V. E. (2005). Isolation and characterisation of an equol‐producing mixed microbial culture from a human faecal sample and its activity under gastrointestinal conditions. Archives of Microbiology, 183(1), 45–55. 10.1007/s00203-004-0747-4 15578160

[fsn32840-bib-0003] Fatima, A. , Khan, M. S. , & Ahmad, M. W. (2020). Therapeutic potential of equol: A comprehensive review. Current Pharmaceutical Design, 26(45), 5837–5843. 10.2174/1381612826999201117122915 33208061

[fsn32840-bib-0004] Gao, Y. N. , Hao, Q.‐H. , Zhang, H.‐L. , Zhou, B. , Yu, X.‐M. , & Wang, X.‐L. (2016). Reduction of soy isoflavones by use of *Escherichia coli* whole‐cell biocatalyst expressing isoflavone reductase under aerobic conditions. Letters in Applied Microbiology, 63(2), 111–116. 27227796 10.1111/lam.12594

[fsn32840-bib-0005] Gou, Z. , Jiang, S. , Zheng, C. , Tian, Z. , & Lin, X. (2015). Equol Inhibits LPS‐induced oxidative stress and enhances the immune response in chicken HD11 macrophages. Cellular Physiology and Biochemistry, 36(2), 611–621. 10.1159/000430124 25997976

[fsn32840-bib-0006] Guo, Y. , Zhao, L. , Fang, X. , Zhong, Q. , Liang, H. , Liang, W. , & Wang, L. (2021). Isolation and identification of a human intestinal bacterium capable of daidzein conversion. FEMS Microbiology Letters, 368(8), 1–7. 10.1093/femsle/fnab046 33930123

[fsn32840-bib-0007] Hu, X. , Liu, L. , Chen, D. , Wang, Y. , Zhang, J. , & Shao, L. (2017). Co‐expression of the recombined alcohol dehydrogenase and glucose dehydrogenase and cross‐linked enzyme aggregates stabilization. Bioresource Technology, 224, 531–535. 10.1016/j.biortech.2016.10.076 27838320

[fsn32840-bib-0008] Hwang, J. , Wang, J. , Morazzoni, P. , Hodis, H. N. , & Sevanian, A. (2003). The phytoestrogen equol increases nitric oxide availability by inhibiting superoxide production: An antioxidant mechanism for cell‐mediated LDL modification. Free Radical Biology and Medicine, 34(10), 1271–1282. 10.1016/S0891-5849(03)00104-7 12726915

[fsn32840-bib-0009] Ishiwata, N. , Melby, M. K. , Mizuno, S. , & Watanabe, S. (2009). New equol supplement for relieving menopausal symptoms: Randomized, placebo‐controlled trial of Japanese women. Menopause, 16(1), 141–148. 10.1097/gme.0b013e31818379fa 19131846

[fsn32840-bib-0010] Kim, E. Y. et al. (2014). Equol induces mitochondria‐mediated apoptosis of human cervical cancer cells. Anticancer Research, 34(9), 4985–4992. 25202081

[fsn32840-bib-0011] Kim, M. et al. (2009). Stereospecific biotransformation of dihydrodaidzein into (3S)‐equol by the human intestinal bacterium Eggerthella strain Julong 732. Applied and Environment Microbiology, 75(10), 3062–3068. 10.1128/AEM.02058-08 PMC2681664 19304836

[fsn32840-bib-0012] Lee, P. G. et al. (2016). P212A mutant of dihydrodaidzein reductase enhances (s)‐equol production and enantioselectivity in a recombinant *Escherichia coli* whole‐cell reaction system. Applied and Environment Microbiology, 82(7), 1992–2002. 10.1128/AEM.03584-15 PMC4807523 26801575

[fsn32840-bib-0013] Lee, P.‐G. , Kim, J. , Kim, E.‐J. , Lee, S.‐H. , Choi, K.‐Y. , Kazlauskas, R. J. , & Kim, B.‐G. (2017). Biosynthesis of (‐)‐5‐hydroxy‐equol and 5‐hydroxy‐dehydroequol from soy isoflavone, genistein using microbial whole cell bioconversion. ACS Chemical Biology, 12(11), 2883–2890. 10.1021/acschembio.7b00624 28985044

[fsn32840-bib-0014] Lee, P.‐G. , Lee, S.‐H. , Kim, J. , Kim, E.‐J. , Choi, K.‐Y. , & Kim, B.‐G. (2018). Polymeric solvent engineering for gram/liter scale production of a water‐insoluble isoflavone derivative, (S)‐equol. Applied Microbiology and Biotechnology, 102(16), 6915–6921. 10.1007/s00253-018-9137-8 29948112

[fsn32840-bib-0015] Lee, W.‐H. , Kim, M.‐D. , Jin, Y.‐S. , & Seo, J.‐H. (2013). Engineering of NADPH regenerators in *Escherichia coli* for enhanced biotransformation. Applied Microbiology and Biotechnology, 97(7), 2761–2772. 10.1007/s00253-013-4750-z 23420268

[fsn32840-bib-0016] Li, H. et al. (2018). To construct an engineered (S)‐equol resistant *E. coli* for in vitro (S)‐equol production. Frontiers in Microbiology, 9, 1182. 29915570 10.3389/fmicb.2018.01182 PMC5994542

[fsn32840-bib-0017] Liu, L. , Yang, H. , Shin, H.‐d. , Chen, R. R. , Li, J. , Du, G. , & Chen, J. (2013). How to achieve high‐level expression of microbial enzymes: Strategies and perspectives. Bioengineered, 4(4), 212–223. 10.4161/bioe.24761 23686280 PMC3728192

[fsn32840-bib-0018] Lu, C. , Gao, R. , Zhang, Y. , Jiang, N. , Chen, Y. , Sun, J. , Wang, Q. , Fan, B. , Liu, X. , & Wang, F. (2021). S‐equol, a metabolite of dietary soy isoflavones, alleviates lipopolysaccharide‐induced depressive‐like behavior in mice by inhibiting neuroinflammation and enhancing synaptic plasticity. Food & Function, 12(13), 5770–5778. 10.1039/D1FO00547B 34038497

[fsn32840-bib-0019] Matthies, A. , Blaut, M. , & Braune, A. (2009). Isolation of a human intestinal bacterium capable of daidzein and genistein conversion. Applied and Environment Microbiology, 75(6), 1740–1744. 10.1128/AEM.01795-08 PMC2655447 19139227

[fsn32840-bib-0020] Mayo, B. , Vazquez, L. , & Florez, A. B. (2019). A bacterial metabolite from the daidzein isoflavone and its presumed beneficial health effects. Nutrients, 11(9), 2231. 10.3390/nu11092231 PMC6770660 31527435

[fsn32840-bib-0021] Muthyala, R. S. , Ju, Y. H. , Sheng, S. , Williams, L. D. , Doerge, D. R. , Katzenellenbogen, B. S. , Helferich, W. G. , & Katzenellenbogen, J. A. (2004). Equol, a natural estrogenic metabolite from soy isoflavones: Convenient preparation and resolution of R‐ and S‐equols and their differing binding and biological activity through estrogen receptors alpha and beta. Bioorganic & Medicinal Chemistry, 12(6), 1559–1567. 10.1016/j.bmc.2003.11.035 15018930

[fsn32840-bib-0022] Ozasa, K. , Nakao, M. , Watanabe, Y. , Hayashi, K. , Miki, T. , Mikami, K. , Mori, M. , Sakauchi, F. , Washio, M. , Ito, Y. , Suzuki, K. , Wakai, K. , & Tamakoshi, A. (2004). Serum phytoestrogens and prostate cancer risk in a nested case‐control study among Japanese men. Cancer Science, 95(1), 65–71. 10.1111/j.1349-7006.2004.tb03172.x 14720329 PMC11160038

[fsn32840-bib-0023] Rowland, I. , Gibson, G. , Heinken, A. , Scott, K. , Swann, J. , Thiele, I. , & Tuohy, K. (2018). Gut microbiota functions: Metabolism of nutrients and other food components. European Journal of Nutrition, 57(1), 1–24. 10.1007/s00394-017-1445-8 PMC5847071 28393285

[fsn32840-bib-0024] Schröder, C. , Matthies, A. , Engst, W. , Blaut, M/ , & Braune, A. (2013). Identification and expression of genes involved in the conversion of daidzein and genistein by the equol‐forming bacterium Slackia isoflavoniconvertens. Applied and Environment Microbiology, 79(11), 3494–3502. 10.1128/AEM.03693-12 PMC3648055 23542626

[fsn32840-bib-0025] Shimada, Y. , Takahashi, M. , Miyazawa, N. , Abiru, Y. , Uchiyama, S. , & Hishigaki, H. (2012). Identification of a novel dihydrodaidzein racemase essential for biosynthesis of equol from daidzein in Lactococcus sp. strain 20–92. Applied and Environment Microbiology, 78(14), 4902–4907. 10.1128/AEM.00410-12 PMC3416379 22582059

[fsn32840-bib-0026] Tsuji, H. , Moriyama, K. , Nomoto, K. , Miyanaga, N. , & Akaza, H. (2010). Isolation and characterization of the equol‐producing bacterium *Slackia* sp. strain NATTS. Archives of Microbiology, 192(4), 279–287. 10.1007/s00203-010-0546-z 20237913

[fsn32840-bib-0027] Yang, S. , Zhu, S.‐F. , Zhang, C.‐M. , Song, S. , Yu, Y.‐B. , Li, S. , & Zhou, Q.‐L. (2012). Enantioselective iridium‐catalyzed hydrogenation of α‐arylcinnamic acids and synthesis of (S)‐equol ‐ ScienceDirect. Tetrahedron, 68(26), 5172–5178.

